# Identifying recombinants in human and primate immunodeficiency virus sequence alignments using quartet scanning

**DOI:** 10.1186/1471-2105-10-126

**Published:** 2009-04-27

**Authors:** Philippe Lemey, Martin Lott, Darren P Martin, Vincent Moulton

**Affiliations:** 1 Rega Institute, Katholieke Universiteit Leuven, Minderbroedersstraat 10, 3000 Leuven, Belgium; 2 School of Computing Sciences, University of East Anglia, NR4 7TJ, Norwich, UK; 3 Institute of Infectious Disease and Molecular Medicine, Faculty Of Health Sciences, University of Cape Town, Observatory 7925, South Africa

## Abstract

**Background:**

Recombination has a profound impact on the evolution of viruses, but characterizing recombination patterns in molecular sequences remains a challenging endeavor. Despite its importance in molecular evolutionary studies, identifying the sequences that exhibit such patterns has received comparatively less attention in the recombination detection framework. Here, we extend a quartet-mapping based recombination detection method to enable identification of recombinant sequences without prior specifications of either query and reference sequences. Through simulations we evaluate different recombinant identification statistics and significance tests. We compare the quartet approach with triplet-based methods that employ additional heuristic tests to identify parental and recombinant sequences.

**Results:**

Analysis of phylogenetic simulations reveal that identifying the descendents of relatively old recombination events is a challenging task for all methods available, and that quartet scanning performs relatively well compared to the triplet based methods. The use of quartet scanning is further demonstrated by analyzing both well-established and putative HIV-1 recombinant strains. In agreement with recent findings, we provide evidence that the presumed circulating recombinant CRF02_AG is a 'pure' lineage, whereas the presumed parental lineage subtype G has a recombinant origin. We also demonstrate HIV-1 intrasubtype recombination, confirm the hybrid origin of SIV in chimpanzees and further disentangle the recombinant history of SIV lineages in a primate immunodeficiency virus data set.

**Conclusion:**

Quartet scanning makes a valuable addition to triplet-based methods for identifying recombinant sequences without prior specifications of either query and reference sequences. The new method is available in the VisRD v.3.0 package .

## Background

Investigating the molecular footprint of recombination in viral gene sequences is a multifaceted discipline. Such studies encompass statistical testing for the occurrence of recombination [e.g. [1,2]], inferring the rate of recombination in a population, identifying parental and recombinant sequences [e.g. [3,4]], and mapping breakpoints in mosaic genomes [e.g. [5,6]]. Various recombination detection methods have been developed that generally focus on one or a subset of these tasks [7] or attempt to address increasing levels of complexity [e.g. [8]]. Most popular methods to investigate viral recombination patterns in a sequence alignment use a graphical sliding window approach to either scan the similarity of a query sequence against a set of parental (or reference) sequences, e.g. RIP [9] and SIMPLOT [6], or to compare the phylogenetic branching patterns of the query sequence relative to the parental sequences along the genome, e.g. BOOTSCAN [10]. However, Martin *et al. *(2005) noted that the proper use of these methods often crucially depends on the prior identification of a suitable set of non-recombinant parental sequences (or 'pure' lineages), and, indeed, inclusion of recombinant reference sequences can result in flawed interpretation.

In HIV-1, for example, subtype G has been classified as a pure subtype for several years, but detailed analyses have recently shown that this strain most likely evolved following recombination between one lineage classifiable as the so-called circulating recombinant form CRF02_AG and another belonging to the 'pure' (i.e. non inter-subtype recombinant) lineage subtype J [11]. CRF02_AG on the other hand, was found to be a 'pure' subtype and its classification as a circulating recombinant form (CRF) could be attributed to the inclusion of subtype G as a reference sequence in the analysis. In this case, the sampling history of subtypes and CRFs has caused a misinterpretation of the evolutionary history of HIV-1 group M: the parental subtype J complete genome was not available at the time subtype G was completely sequenced, so a recombinant pattern for subtype G could not be clearly established (although some ambiguous relationships with subtype A and CRF01_AE were noted) [12]. Therefore, subtype G was assigned as a reference strain in the subsequent analysis of CRF02_AG, resulting in a misleading BOOTSCAN profile that suggested a mosaic genome for CRF02_AG [12].

The CRF02_AG example demonstrates that the 'query versus reference' approach will only be suitable to identify recombinants if valid reference sequences can be assigned. Such prior specification is essential for many methods, including dedicated HIV-1 methods and web servers [e.g. [13,14]]. An additional shortcoming of graphical sliding window based methods, like BOOTSCAN, is that they commonly lack a formal test for recombination. Although bootstrap support values are well studied in phylogenetics, there exists no clear statistical basis to conclude significance evidence for recombination based on bootstrap variability across genome regions. In fact, by assessing significance conditional on reference sequences and crossover points that maximize the same test statistic, the BOOTSCAN approach falls into a sequential testing trap [15].

To avoid the problem of assigning appropriate reference sequences, exploratory methods have been proposed that consider every sequence as a potential recombinant or parent in a scanning procedure. RECSCAN, for example, is a modified BOOTSCAN algorithm that checks all combinations of three sequences (triplets) for changing nearest neighbor relationships. If different relationships are observed with bootstrap support above a user-specified value, a test of recombination is applied to the triplet [16]. With appropriate multiple testing correction, a wide array of test approaches can be applied to all triplets to identify those combinations that show evidence for recombination, including also substitution distribution methods like maximum chi^2 ^and GENECONV [2,17]. None of these triplet exploration procedures, however, identify the mosaic sequence within a triplet that shows evidence for recombination. To tease apart recombinant from parental descendent sequences, Heath *et al*. (2006) proposed a series of additional heuristic tests that examine which sequence relationships change the most across a recombination breakpoint. A weighted consensus of these tests is eventually responsible for calling the recombinant sequence. Unfortunately, this weighting scheme is an arbitrary choice that is currently based on how accurately HIV-1 CRFs are identified as mosaic genomes. Given the problems concerning CRFs and subtype reference sequences discussed above, approaches that do not require empirical decisions would be a useful alternative for recombinant identification. Other methods have been proposed for recombinant identification [18], but accurate prior classification of sequences remains essential for their performance. Moreover, while the performance of different methods to detect the presence of recombination has been extensively evaluated [19,20], no such efforts have been undertaken to investigate how well methods perform in teasing out recombinant sequences.

Here, we present an alternative approach to detect recombinants without prior identification of non-recombinant reference strains. We employ quartet-trees to rapidly scan for phylogenetic inhomogeneity along a sequence alignment and demonstrate how this information can also be employed to identify those sequences responsible for detectable recombination signals. Using simulated data sets we evaluate different quartet incongruence measures, different approaches to assess the significance of various recombination detection statistics, and compare their performance at identifying recombinant sequences with that of triplet-based methods. Finally, the usefulness of these methods in investigating recombination is demonstrated at different scales of primate immunodeficiency virus (PIV) evolution, including both HIV and SIV.

## Methods

We describe the method developments in several subsections. The procedure we present here is an extension of a previously developed visual recombination detection (VisRD) method. We propose a scanning method that evaluates whether all combinations of four sequences (quartets) in a sequence alignment jointly provide evidence for recombination. When this is the case, we rank taxa or groups of taxa according to their contribution to this recombination signal. The method can be employed to sequentially prune putative recombinants until no significant recombination evidence can be found in the alignment. We start by briefly explaining the previously developed visual recombination detection method based on quartet scanning. Following the description of a novel quartet mapping approach based on a distance-based method, we present measures for phylogenetic inhomogeneity of quartets, ranking measures for taxa and groups of taxa, and a global test statistic for recombination. We then describe how null distributions can be obtained for this test statistic. In addition to the VisRD method, we briefly explain alternative triplet approaches used in our comparisons. We conclude the methods section by providing details on the simulated and empirical data used in this study.

### Visual Recombination Detection

**Figure 1:**
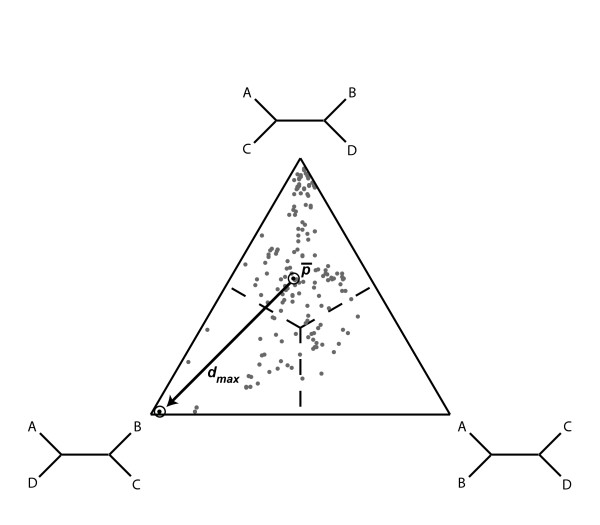
**Representation of the quartet-ranking principle**. In the quartet-mapping triangle, each dot represents the relative support for the three unrooted topologies [25]. The quartet-mapping triangle depicted here summarizes the support across all windows in the scanning procedure. The mean position for all quartets is indicated by  and the maximum distance from the mean position is indicated by an arrow (*d*_*max*_). This example is based on the quartet with the highest *d*_*max *_in the 'pure' subtypes of the CRF03_AB data set (see Sequence data).

**Figure 2:**
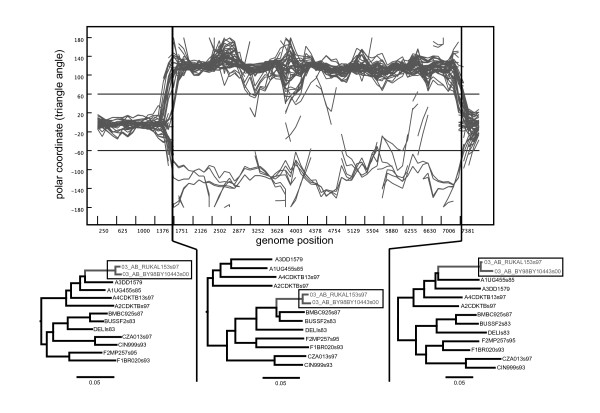
**Highway plot and phylogenies for the CRF03_AB data set**. In this plot, the horizontal axis represents the alignment sites, whereas the vertical axis indicates changes of topology and branch length of the inferred quartet trees along the alignment [22]. The three 'lanes', which lie between the three horizontal lines in the plot, reflect the fact that a quartet tree can have three possible topologies. Each curve or 'trajectory' in the plot represents changes in the inferred quartet tree along the alignment for a particular quartet of sequences. Only the trajectories for the 50 top-ranking quartets are shown. Maximum likelihood trees for the three separate gene regions, separated by the breakpoints inferred from the quartet trajectory changes, are shown below the highway plot. The recombinant strains are indicated with a rectangle.

The VisRD method is designed to visually inspect sequence alignments for recombination events [21,22]. VisRD works by computing quartets, or unrooted phylogenetic trees on four leaves, for each possible four-taxa set. More specifically, at each window (*i*) in an alignment, a support *s*_*i *_for each of the three possible quartet topologies *T*_*i *_is computed using a statistical geometry approach [23]. Essentially, the support value *s*_*i *_is computed by summing the number of site patterns that support *T*_*i*_. Following the quartet mapping approach [24] [a generalization of likelihood mapping, [25]], a relative support is then derived that is defined as s_*i*_/(s_1_+s_2_+s_3_). Subsequently, the relative supports for each quartet topology are summarized in a quartet-mapping triangle; in this triangle, each quartet (in this case, a single sliding window partition of a four sequence alignment) is represented as a point (Figure 1), whose co-ordinates reflect the support for each of the quartet topologies. The corners of the triangle represent the three fully resolved topologies for four taxa. A point located close to such a corner reflects a high support for this particular topology; the three relative support values are represented by the lengths of the perpendiculars from the point to the triangle sides [25]. By considering all of the windows along the alignment, a trajectory in the triangle is then computed between the points representing consecutive alignment partitions, the rationale being that if recombination has occurred then points will follow highly variable trajectories. These trajectories are then filtered to reduce noise, and depicted in a highway plot (see for example, Figure 2), which can be visually inspected for recombination signals. In the highway plot, the horizontal axis represents the sites in the alignment, whereas the three lanes represent the regions supporting different topologies (delineated by the dashed lines in the triangle of Figure 1). Mapping points from a quartet triangle onto the vertical axis of the highway plot is achieved by the polar coordinates of the points; the most variable trajectories between all the points are filtered out to make up the highway plot [22].

### Distance-based quartet mapping

As indicated above, the quartet mapping approach used in VisRD is based on statistical geometry [22,23]. Although this eases computation [compared to likelihood mapping, [25], for example], it does not take into account the complexities of nucleotide or amino acid substitution patterns. To address this shortcoming in the new version of VisRD, but still resort to fast quartet computation, we have implemented a distance-based minimum evolution (ME) approach to quartet-mapping. Briefly, branch lengths for each quartet topology are obtained using least-squares solutions [26], and the relative support for topology *T*_*i *_is given by , where *ME *is the sum of all positive branch lengths. For nucleotide sequences, the Jukes-Cantor, Kimura-two-parameter, Felsenstein '84 (F84) and Tamura-Nei substitution models were implemented.

For protein sequences, we implemented a protein distance estimator that uses a logarithmic correction of observed divergence based on the alignment score derived from empirical transition probability matrices [27]. This estimator provides accurate and robust protein distances and can be easily adjusted to use any empirical log odds substitution matrix. In addition to the BLOSUM62 model, we also included HIV-specific matrices [28] and constructed a matrix specifically for analyses of more divergent PIV sequences. For the latter, we downloaded the available amino acid alignments for the Gag, Pol, Env and Nef proteins from the HIV database , including 67, 86, 84 and 68 sequences respectively. Ambiguously aligned regions were deleted and phylogenetic trees were inferred using PhyML [29]. The stochastic amino acid substitution model was inferred using a maximum likelihood phylogenetic approach that estimates the 190 evolutionary rates, defining the general time reversible model of amino-acid substitution, jointly from a set of sequence alignments. Finally, a similarity matrix with an expected sequence dissimilarity of 38% was generated as previously described using HyPhy [28,30].

Nucleotide sequence alignments were analyzed using the F84 model and a sliding window with a window size of 500 bp and a step size of 40 bp. Amino acid sequence data was analyzed using the PIV log odds matrix, with a window size of 150 aa and a step size of 10 aa. Since nucleotide sequence alignments can include a number of ambiguous characters, particularly for a population of virus sequences, we also employed a random resolution of ambiguous sites to one of their possible states based on the nature of the ambiguity. For example, a site denoted with the IUBMB symbol 'M' could be either an 'A' or 'C' nucleotide; each is picked with equal probability when distances are computed using one of the above models.

### Taxon ranking, group ranking and test statistics

We now introduce methods for ranking taxa according to their phylogenetic variability, and we derive a test statistic for the presence of recombination in the global data set. The purpose of the ranking is to identify the most likely recombinants in case the sequence alignment set contains significant evidence for recombination. The ranking of taxa is achieved using measures of variability for quartets that contain these taxa. The first measure of quartet trajectory variability computes a score for each quartet *q *by determining the average position of the corresponding point *p*_*i*_(*q*) in the triangle across all alignment windows, (*q*), and then computing the maximum distance of any point from this position across all windows (*d*_max _(*q*) = max|*p*_*i*_(*q*) - (*q*)|, Figure 1). This is repeated for all quartets, and, for each taxon *t*, a rank (*r*_*t*_) is then computed as the average *d*_max _value taken over all quartets that include *t*. In particular, defining *Q*_*t *_as being the set of quartets that involve taxon *t*, and letting |*Q*_*t*_| denote the size of *Q*_*t*_, the rank *r*_*t *_is given by

(1)

We also introduce a grouping model as an extension to the four taxa model in VisRD, allowing any number of groups, perhaps representing well-established virus subtypes or genotypes. In particular, for a pre-specified group of taxa *g*, a group rank (*r*_*g*_) is computed by taking the average *d*_max _value over all taxa in group *g*, and over all quartets *q*_*t *_that include *t*, that is,

(2)

Using the group rank (*r*_*g*_), we can focus on recombinant events between pre-specified groups while alleviating some of the computational burden of the quartet scanning procedure. The mean taxon ranking  and mean group ranking , which quantify the phylogenetic incongruence in the alignment, are then taken to be the average of these values taken over all taxa and all groups, respectively.

Alternatively, a rank for each quartet can be calculated based on the total distance covered by the trajectory for each quartet (*d*_tot _(*q*)) or the total distance from each point to the average position of all points in the trajectory (*d*_av _(*q*)). By again computing the average *d*_tot _or *d*_av _value taken over all *n *quartets *q*_*t *_that include *t*, and averaging over a group, we can obtain a taxon ranking and group ranking (substituting *d*_max _by *d*_tot _and *d*_av _in (1) and (2) respectively).

The taxon and group ranking values (*r*_*t *_and *r*_*g*_) are computed using all quartet trajectories (filtering is only considered in constructing the highway plot). The ranking values can be used to assess the contribution to phylogenetic inhomogeneity of individual taxa and groups respectively (i.e. they can be used to identify recombinant sequences), whereas the mean taxon and group ranking values ( and ) can be individually used as statistics for detecting recombination in a sequence alignment (see *Significance*). To identify all plausible recombinants in a sequence alignment using the quartet scanning procedure, we follow a sequential deletion procedure. When significant evidence of recombination is detected using  or , the top-ranked sequence is considered as a putative recombinant and removed from the alignment. We repeat this until no significant evidence of recombination remains in the data set. Because the absolute values of *d*_*max*_, and hence *r*_*t *_and *r*_*g*_, are sensitive to the evolutionary models and topology evaluation criteria used, we report the ranking values as percentages of the highest ranking taxon or group.

### Significance

We evaluate three different ways to generate a null distribution (absence of recombination) for the observed test statistics  and  derived from the taxon-ranking procedure: (i) Monte Carlo simulation (MC-simulation), (ii) permutation, and (iii) 'redistribution' of alignment columns. (i) For the MC-simulation procedure, maximum likelihood phylogenetic trees are reconstructed using PhyML [29], employing the general time-reversible substitution model (GTR) and gamma-distributed rate variation among sites. Because a null distribution needs to be generated under the hypothesis of 'no recombination', we simulate replicate data sets using Seq-Gen [31] down a single phylogeny reconstructed from the complete alignment. The same models and parameters were used as obtained by the maximum likelihood tree inference. (ii) Since taxon ranking is based on topological incongruence, we also use a permutation procedure that homogenizes any phylogenetic incongruence along the genome (under the null hypothesis of no recombination, the sliding window analysis should be invariant to permuting sites as all sites share the same history). This is achieved by random shuffling of the original alignment sites to produce replicate data sets. (iii) To accommodate varying phylogenetic signal along the genome, which is often observed in real data due to different degrees of conservation within the sequence, we also use a permutation procedure that mimics the variability distribution of the alignment columns ('redistribution'). Essentially, this procedure reshuffles alignment columns according to their Shannon entropy score [32]. Replicate data sets are constructed by randomly drawing for each alignment position an alignment column with the same entropy score.

We use these three procedures to generate 100 replicate data sets each, so as to compute null distributions for our test statistics ( or ). A Monte Carlo *p*-value is then estimated by counting the proportion of times the  or  statistic on a replicate data set is larger than the value of  or  observed for the original alignment. If the null hypothesis is true, this proportion is expected to be 0.5. Such a *p*-value represents the probability of obtaining a result at least as extreme as observed for the original alignment in the absence of recombination.

### RDP3 recombinant detection

The RDP3 software provides access to multiple recombination signal detection methods that can be used in conjunction with one another [33]. The methods (CHIMAERA, Maximum chi^2^, RDP, RECSCAN and Geneconv, [2,17,20,34]) in RDP3 search for recombination signals within sequence triplets sampled from an input alignment. When a recombination signal is detected, the member of the sequence triplet that is the recombinant is identified using a weighted consensus of a series of fifteen heuristic tests [mostly described in [35]]. These tests fall into four main categories: (i) Phylogenetic profile based (4 tests; [35,36]); (ii) branch pruning and re-grafting based (2 tests; [35,37,38]); (iii) multiple recombination signal pattern analysis based (3 tests; [35]); and (iv) simple distance-based metrics (5 tests) that, for example, compare pair-wise genetic, and phylogenetic tree distances of different subsets of sequences within the alignment on opposite sides of the detected recombination breakpoint position. These latter tests collectively identify the recombinant by determining which sequence(s) within an alignment have relationships that change the most across a recombination breakpoint. The weighting scheme used in RDP3 to combine the results of all 15 tests into a single consensus score is based on how accurately the individual tests identified HIV-1M inter-subtype recombinants (circulating recombinant forms 1 through 16) from amongst a background of supposedly non-recombinant HIV-1M subtype sequences.

### Simulation studies

Two different simulation studies were undertaken: (i) to evaluate how accurately the methods detected specific recombinant sequences, and (ii) to evaluate the overall power of methods at detecting recombination signals within sequence alignments (irrespective of which sequences are responsible for these signals).

**Figure 3:**
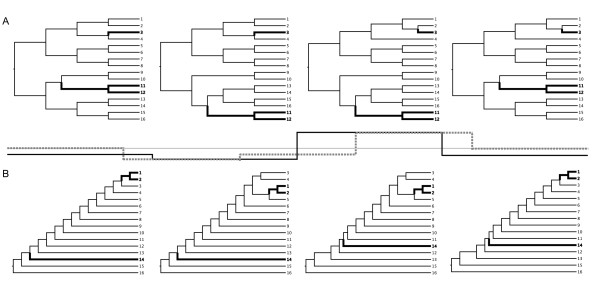
**Phylogenetic simulation of recombinant sequences**. Sequence data sets were simulated according to (a) symmetric and (b) asymmetric tree topologies. Each tree represents a single partition of 1000 nucleotides. In the symmetric combination, this results in a single breakpoint mosaic pattern for taxon 3 and a shared dual breakpoint mosaic pattern for taxon 11 and 12. In the asymmetric combination, taxon 1 and 2 share a relative recent recombination event with two breakpoints, while taxon 14 is a descendent of an older single breakpoint recombination event. In between both sets of trees, the relative rate profiles are plotted relative to 1 (thin line): the black line pattern represents rate variation correlated with the breakpoint positions (0.75, 0.50, 2.00 and 0.75), while the grey dashed line represents rate variation for partitions of 800 bp (1.0, 0.50, 0.75, 2.00 and 0.75).

For (i), sequence data sets were simulated using Seq-Gen [31] according to different trees for different data partitions (Figure 3). Data sets were simulated using both a series of symmetric and asymmetric trees. In the symmetric setup, taxon 3 is a relatively recent recombinant with a single breakpoint, while taxa 11 and 12 share a recombination event deeper in the tree resulting in two breakpoints for each sequence. In the asymmetric setup, taxa 1 and 2 share a relatively recent recombination event with two breakpoints, while taxon 14 represents an older recombinant with a single breakpoint. A thousand nucleotides were evolved on each of these trees using the Hasegawa-Kishino-Yano nucleotide substitution model with gamma-distributed rate variation (transition/transversion ratio = 4.0, alpha = 1.0), resulting in a total sequence length of 4000 nucleotides. To simulate data sets with different degrees of diversity, we employed total tree depths of 0.5, 1.0 and 1.5 substitutions per site (1.0 is close to the general tree depth of HIV-1 group M data sets, see below). In addition to rate variation among sites, we also investigated the impact of rate variation among different alignment regions by employing different relative rates for each 1000 nucleotide partition (relative rates: 0.75, 0.50, 2.00 and 0.75 respectively) and different relative rates for 800 nucleotide partitions (1.0, 0.5, 0.75, 2.0, 0.75). For each treatment (either symmetric or asymmetric tree setup, in combination with different tree lengths, and with or with rate variation among partitions), 100 data sets were simulated.

For (ii), to evaluate power and false positive rates in detection of recombination signals, we examined simulated data sets previously generated for comparing the performance of 14 recombination detection methods [20]. These data sets are now often used to evaluate recombination detection power and false positives of new recombination detection programs [16,39].

### Empirical data

The HIV-1 group M alignments analyzed here were derived from a full genome alignment provided by the HIV database  and manually edited using Se-Al . The accession numbers of the included sequences are listed in Additional File 1. Two data sets were used in our analyses: a CRF03_AB data set (including 4 A's, 2 B's, 2 C's, 1 D, 2F's and 2 CRF03_AB's) and a larger CRF02_AG data set (including 4 A's, 4 B's, 4 C's, 4 D's, 4 F's, 4 G's, 2 H's, 2 J's, 2 K's and 4 CRF02_AG's). The HIV-1 group M alignments had nucleotide diversities ranging from 0.17 substitutions/site to 0.19 substitutions/site, which roughly corresponds to θ = 200 in the coalescent-based simulations. The SIV data set, previously analyzed for recombination by Bailes *et al. *(2003), includes protein sequences representative of eight major SIV clades. Their accession numbers are also listed in Additional File 1.

Bootstrapped phylogenetic trees were reconstructed using PhyML [29], as described above (see Significance) and network analyses were performed using the Neighbor-net method [40] implemented in SplitsTree [41]. To represent robust network-like relationships, the Neighbor-nets only display the splits that were present in 75% of 1000 bootstrapped replicates. Intrasubtype recombination was analyzed using GARD method, available at [39]. Tree topology tests were performed using Tree-Puzzle [42] and Consel [43].

## Results

We present the results of three main analysis sections: (i) simulations to evaluate the performance of different statistics and measures of significance for the quartet scanning method, and simulation studies to compare quartet scanning with other recombination detection methods in terms of both detecting recombination signals and identifying recombinant sequences; (ii) an analysis of HIV-1 group M inter- and intra-subtype recombination; and (iii) recombinant identification using amino acid data from more divergent PIV lineages.

### Simulated data analysis

**Figure 4:**
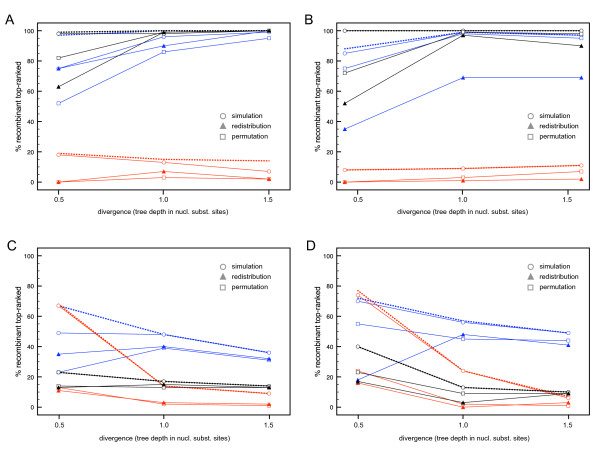
**Evaluating different quartet scanning statistics and significance assessments**. The percentages of correctly top-ranked recombinants for three different statistics and three different approaches to evaluate significance are summarized for simulated data in the absence (b and d) and presence (a and c) of rate variation among partitions. The upper graphs (a and b) and the lower graphs (c and d) summarize the results for the symmetric and asymmetric trees respectively. Dashed lines represent the frequency by which a recombinant is correctly ranked on top, independent of whether significant evidence for recombination can be detected in the replicate set. The full lines represent the same frequency, but now supported with significant evidence for recombination in the replicate set. The different statistics, *d*_max_, *d*_tot _and *d*_av_, are represented by blue, red and black lines/symbols respectively. Simulation, permutation and redistribution to generate null distributions for these test statistics are represented by open circles, open squares and filled triangles respectively. The results are shown for simulations using three different tree depths (0.5, 1.0 and 1.5 substitutions per site).

To establish the most powerful test statistic and significance assessment for the distance-based quartet scanning procedure, we analyzed data sets simulated on both symmetric and asymmetric trees, which generates three recombinant sequences per data set (Figure 3). To restrict the amount of computation in these simulation analyses, we focus on the first sequence ranked on top by the quartet scanning approach and perform sequential deletion whilst significant evidence for recombination can be detected. The results are summarized in Figure 4. Dashed lines represent the frequency by which a recombinant is correctly ranked on top, independent of whether significant evidence for recombination can be detected in the replicate set. The full lines represent the same frequency, but now supported with significant evidence for recombination in the replicate set. The upper graphs summarize the results for symmetric trees (Figure 4a and 4b), while the lower graphs summarize the results for asymmetric trees (Figure 4c and 4d); the graphs on the right present the results for data sets simulated with different relative rates correlated with the alignment partitions (Figure 4b and 4d; see Methods). Overall, recombination detection was more successful in symmetric trees and a correctly identified recombinant was usually a 'recent' recombinant (sequence 3 in the symmetric trees and sequence 1 and 2 in the asymmetric trees; Figure 3). The total trajectory distance (*d*_tot_) appeared to be a poor test statistic for recombination detection; the total distance from the mean position in the quartet triangles (*d*_av_) was most powerful in symmetric trees but the maximum distance from the mean position (*d*_max_) appeared to be the best overall statistic. We thus chose *d*_max _as test statistic for further simulation analyses. Increasing sequence diversity resulted in increased power of recombinant detection in the symmetric trees (especially between tree depths of 0.5 and 1.0 substitutions per site). Somewhat surprisingly, this was usually not the case for analysis of asymmetric trees. In general, simulation led to stronger statistical support than permutation. Permutation, in turn, was more powerful than redistribution. Redistribution also resulted in a markedly lower number of correctly identified recombinants, with statistical support for recombination in the replicate data set, in the presence of rate variation among alignment partitions. The fact that the partitions in between recombination breakpoints coincide with the relative rate partitions in our simulation may be at least partly responsible for this, as sites may not be efficiently permutated across breakpoints in the redistribution procedure.

To assess the performance of the distance-based quartet scanning method as a statistical test for recombination signal, rather than a method for recombinant identification, we also reanalyzed nucleotide data simulated under varying levels of divergence and recombination originally presented in [20] and compared this with other methods (see Additional File 2 for a detailed presentation of the results). In general, these analyses revealed that the performance of our test is comparable to similar methods, like RECSCAN [16], but these are less powerful than the MAXIMUM CHI^2 ^method [44], which has been reported as one of the most powerful nonparametric recombination detection methods.

**Figure 5:**
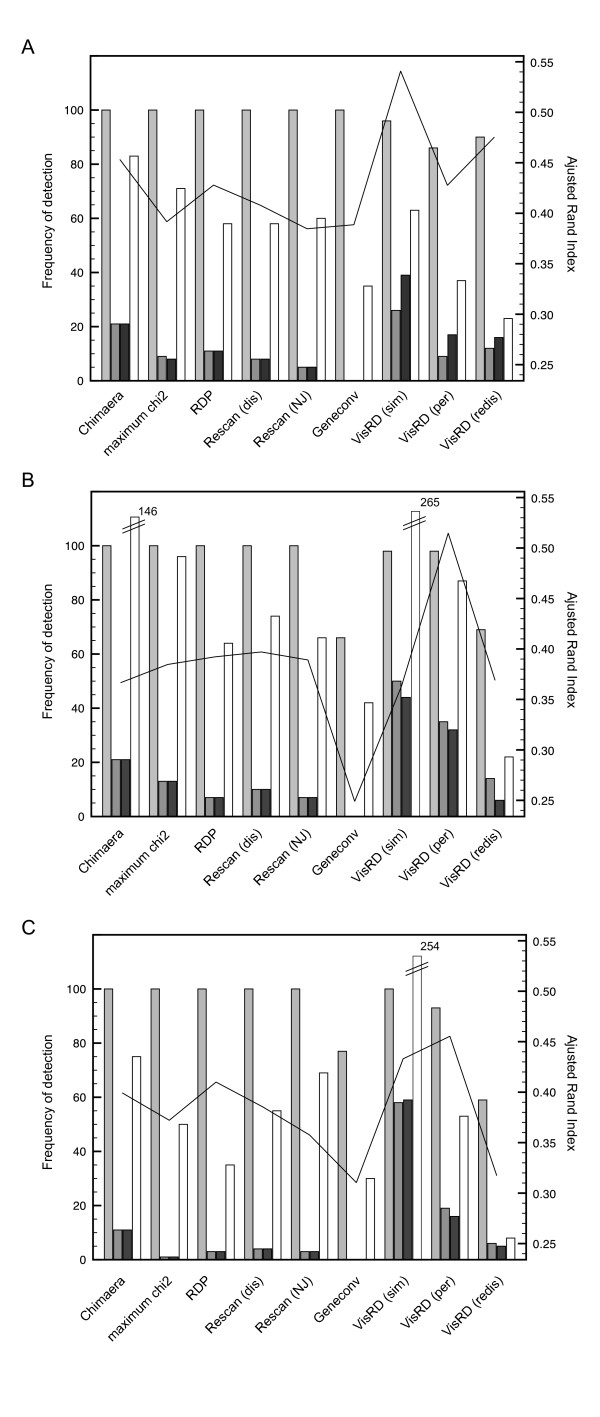
**Performance of triplet-based and quartet-based methods in recombination identification**. Frequency of correctly identified recombinants and false positives in simulated data sets in the absence (a) and presence of rate variation among 1000 nucleotide partitions (b) and 800 nucleotide partitions (c). The latter rate variation scheme is not correlated with the breakpoint distribution. The four bars per method represent the frequency by which taxon 3 (light grey), taxon 11 (dark grey) and taxon 12 (black) were correctly identified as recombinants and the number of incorrectly identified recombinants (white) in 100 simulated data sets of 16 taxa. For RECSCAN, either raw pairwise distances (dis) or neighbor-joining (NJ) trees were inferred for each triplet. For the VisRD method, MC-simulation (sim), permutation (per) and redistribution (redis) were tested. The line represents the adjusted Rand index as an overall performance measure (secondary y-axis).

Finally, we compared the quartet scanning with triplet-based approaches and their associated heuristic tests in their ability to identify the mosaic sequences. Because this requires a sequential deletion procedure in VisRD, we restricted the computation by focusing on the data sets simulated using symmetric trees (Figure 3b) and a tree depth of 1.0 substitution per site, similar to the HIV-1 group M data sets we analyze below. Figure 5 summarizes the frequencies with which taxa 3, 11 and 12 were ranked on top at some stage in the sequential deletion procedure, and hence identified as recombinants, by the different approaches, as well as the total number false positives (sequences ranked on top although simulated as non-recombinants) in the absence and presence of rate variation among sequence regions (Figure 5a versus 5b and 5c respectively). The performance of the different methods is evaluated using the adjusted Rand index [secondary y-axis, [45]]. The adjusted Rand index is the Rand index corrected for chance events, which is commonly used to measure the performance of data clustering. In our case, the adjusted Rand index reflects the percentage of correctly identified recombinant and non-recombinant sequences corrected for chance. Therefore, higher values of this measure indicate higher classification accuracy. The methods mainly differ in the frequencies with which taxa 11 and 12 – the sequences descended from a common recombinant ancestor – are identified as recombinants. Without rate variation among partitions, quartet scanning based on simulation achieves the best rate of detection of these recombinants and obtains the highest performance score. Using permutation and redistribution, top-ranked recombinants were still relatively frequently associated with significant evidence for recombination in the alignment, but the lower detection rate of taxon 3 indicates some sensitivity decrease. The presence of rate variation among partitions increases the false positive rate in many methods (Figure 5b); quartet scanning with simulation is particularly sensitive to this. A permutation procedure, however, still achieves good detection rates without an excessive number of false positives. As also suggested by the first set of simulation analyses, redistribution becomes less powerful in detecting recombination signal with the simulated rate variation scheme. To investigate whether this results from inefficient permutation across breakpoints correlated with rate variation, we also performed additional simulations using a rate variation scheme that does not coincide with recombination breakpoints (Figure 5c). Also in this case, redistribution did not prove to be a very powerful approach to assess significance. We therefore restrict significance assessment to simulation and permutation in subsequent analyses of real sequence data.

### HIV-1 intersubtype recombination

We applied the VisRD based recombination detection methods to the HIV-1 group M full coding genome data set which included representative sequences for subtypes A-D, and F and the circulating recombinant form CRF03_AB (see Figure 2). The CRF03_AB sequences have been clearly identified as having descended from a common AB recombinant form that circulated amongst injecting drug users in Russian and Ukrainian cities [46,47]. In the quartet scanning procedure, the sequences were analyzed by grouping them according to their subtype or CRF assignment. This group model only considers quartets composed of sequences from four different pre-specified groups, which allows the analysis to focus exclusively on inter-subtype recombination events while reducing the computational burden. In Figure 2, a highway plot provides a visual summary of the most variable trajectories obtained during the quartet scanning procedure. The three lanes in the plot represent the different topology-supporting regions in a quartet triangle (Figure 1), and the change in support is calculated as a function of the alignment position for each quartet. The highway plot clearly indicates two breakpoints at approximately nucleotide positions 2671 and 8655 (according to the HXB2 numbering). Table 1 lists the results of the taxon-ranking procedure for the different groups in this data set, indicating that quartets containing a CRF03_AB sequence show the highest variability in phylogenetic clustering along the genome based on both *d*_max _and *d*_av_. Evidence for recombination in the data set is statistically supported by both simulation and permutation (*p *< 0.01 for the *d*_max _and *d*_av _statistics). The parental subtype A and B sequences are ranked just below the recombinant group due to the high trajectory variability of quartets including one or both parental subtypes and the recombinants. Even after deletion of the CRF03_AB sequences, there was still significant evidence for recombination; a more in depth investigation of HIV-1 group M recombination is provided below.

**Table 1:** Group ranking based on quartet scanning of the CRF03_AB data set.

Groups	*r*_*g *_(%)	
	*d*_max_	*d*_av_
CRF03_AB (2)	100.00%	100.00%
A (4)	90.03%	90.73%
B (2)	89.34%	90.30%
C (2)	82.59%	82.83%
D (2)	81.23%	82.71%
F (2)	80.31%	81.96%

The group ranking values are listed as percentages of the highest ranked group; these values are shown for the maximum distance (*d*_max_) and total distance (*d*_av_) from the mean position in the quartet-mapping triangle. The number of sequences per group is indicated between brackets

The CRF03_AB example indicates that the  values allow us to test for the presence of recombination while taxon ranking itself indicates the most likely recombinants in the data set without assigning query and parental sequences *a priori*. Together, this represents an ideal approach to evaluate recombination patterns in cases where prior identification of non-recombinant reference strains is problematic, like CRF02_AG [11]. To investigate the CRF02_AG issue and other putative recombination events in HIV-1 group M, we compiled a full genome data set that is more representative of HIV-1 group M diversity. Also for the analysis of this data set, we employed a grouping model for the different subtypes and CRFs. Although the  value for the data set including CRF02_AG indicates the presence of recombination (permutation *p *< 0.01; using *d*_max_), the taxon ranking does not suggest CRF02_AG sequences as most plausible mosaic genomes (Additional File 3, 1^st ^column). In agreement with the recent analysis of Abecasis *et al*. (2007), the 'pure' subtype G sequences are proposed as the most likely recombinants while CRF02_AG and subtype J are suggested as parental lineages because they were ranked right below the putative recombinant. When the subtype G sequences were removed, recombination in the remaining data could not be excluded based on the permutation statistics (permutation *p *< 0.01). This time, subtype K was at the top of the taxon ranking (Additional File 3, 3^rd ^column). It is interesting to note that a recombinant origin for subtype K has been proposed [48], but its designation was not changed since it was already an established subtype in the literature [49].

**Figure 6:**
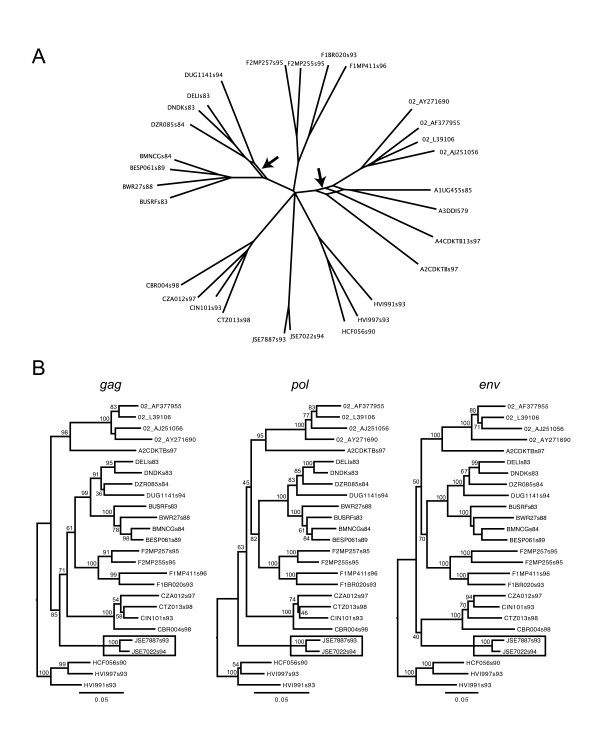
**HIV-1 Neighbor-net and maximum likelihood trees**. (a) Neigbor-net for the HIV-1 group M data set excluding subtype G and subtype K sequences. Only splits are shown that were present in 75% of 1000 bootstrap replicates. Conflicting phylogenetic relationships are indicated with arrows. (b) HIV-1 Maximum likelihood trees for the structural genes *gag*, *pol *and *env*. The numbers at the nodes represent the bootstrap support percentages based on 1000 replicates. The subtype J sequences are indicated with a rectangle.

After excluding both subtype G and K, there was still significant evidence for recombination in the remaining sequences (permutation *p *< 0.01) with subtype A heading the taxon ranking (Additional File 3, 5^th ^column). To our knowledge, subtype A has not been proposed as a intersubtype recombinant before and the recombination signal we detected might result from intra-subtype recombination or intersubtype recombination for particular lineages within subtype A. We investigated this in more detail by removing the grouping model for the different A sub-subtypes and by investigating the evolutionary relationships using Neighbor-nets [40] (Figure 6a). When no grouping for subtype A was used, sub-subtypes A1, A4 and A3 were ranked on top but not sub-subtype A2 (Additional File 3, 7^th ^column). The Neighbor-net indeed demonstrates network-like behaviour among these lineages within subtype A (Figure 6a).

Having removed sub-subtype A1, A4 and A3, our recombination test was still significant (permutation *p *< 0.01) and subtype J was now at the top of the ranking (Additional File 3, 9^th ^column). Although recombination has not been clearly suggested for this subtype, it does indeed cluster differently in each major gene tree of the HIV genome (Figure 6b: branching off after subtype H and CRF02_AG/A2 in *gag*, branching off after subtype H in *pol *and clustering with subtype C in *env*). Further removing of subtype J, there was still significant recombination signal (permutation *p *< 0.01), this time with subtype D heading the ranking (Additional File 3, 11^th ^column). Without the grouping model, three subtype D sequences appeared to be the descendents of a recombinant lineage (Additional File 3, 13^th ^column), which was also confirmed by the Neighbor-net analysis (Figure 6a). The final recombination signal was detected with subtype F heading the ranking, despite a stable clustering of this subtype in the major HIV genes encoding structural proteins (Figure 6b). Recombination remained detectable after deletion of subtype F using the MC-simulation approach, but given the more likely confounding effect of rate variation for MC-simulation and the increasing difficulty of correctly identifying 'deeper' recombinants (both cf. the simulation analysis), we did not explore further taxa deletion. A different order in putative recombinant deletion was suggested after identification of recombination in subtype G, K and subsubtypes A using *d*_av _as a test statistic, and subtype J was not suggested as a recombinant by this procedure (Additional File 4). The intrasubtype recombination detected in subtype A and D in both procedures was confirmed by GARD analysis (data not shown) [39].

We also employed our method to investigate a highly divergent HIV-1 variant from the Democratic Republic of Congo (DRC) [50,51]. Although the DRC strain was positioned differently in phylogenetic trees of different genome regions (similar to subtype J in Figure 6b), it did not cluster with any of the know subtypes and hence it was suggested as a candidate for a new subtype [50]. As reference strains, we included the sequence set for which no significant recombination could be detected (see above, including subtype A2, B, C, DUG114, H, and CRF02_AG). Our taxon-ranking procedure indicated that quartets containing this unclassified variant show the highest phylogenetic variability along the genome (Additional File 5), and this was associated with significant evidence for recombination in the alignment (permutation *p *< 0.01). To confirm alternative clustering patterns for this lineage and to demonstrate significant incongruence despite convincing bootstrap support values, we reconstructed phylogenies for the major genes (*gag*, *pol *and *env*) and performed tree topology tests (Additional file 6). The trees indicate different clustering for the DRC lineage in the three major genes, and despite low bootstrap support for the alternative clustering, tree topology tests consistently indicated significant incongruence. Finally, we also analyzed CRF01_AE sequences, for which only the subtype A lineage is available as closely related parental descendent. The taxon-ranking procedure still revealed significant recombinant signal and implicated this CRF as the lineage responsible for the incongruence (Additional File 7; permutation *p *< 0.01).

### Recombination in primate immunodeficiency virus evolution

Recombination in more divergent simian immunodeficiency viruses (SIVs) was investigated for the amino acid data set of Bailes *et al. *(2003), which includes representatives of eight major SIV lineages for which full-length sequences were available at the that time [52]. Because there are no equidistant SIV clades similar to HIV-1 group M subtypes, we did not apply the grouping model in this case. However, when sister taxa representing one of the eight major lineages were ranked as the top two sequences, we removed both taxa in the subsequent taxon-ranking test. The protein sequences were analyzed using a corrected distance measure based on an amino acid substitution matrix inferred from a larger PIV data set (see Methods), and significance was assessed using permutation. Additional File 8 summarizes the results using the *d*_max _statistic, while the results using *d*_av _are provided in Additional File 9.

The ranking procedure provided evidence for recombination in the SIV sequences (*p *< 0.01). Moreover, in agreement with the findings of a hybrid origin of SIV in chimpanzees [52], SIVcpz was suggested as contributing the most to this recombination signal (Additional File 9, 1^st ^column). However, recombination was also detected during subsequent deletion of several SIV lineages (SIVagm, SIVcol, SIVmnd/l'hoest, SIVsyk/SIVsm/HIV-2), leaving only a single quartet that did not deviate significantly from clonality (*p *= 0.43). There were some differences in the order by which SIVs were identified as recombinants using *d*_av _(see Additional File 9), but the final result was the same.

## Discussion

In this study, we have developed an extension of the visual recombination detection method to rank taxa or groups of taxa according to their contribution to phylogenetic inhomogeneity, and to test whether the overall phylogenetic incongruence has been significantly shaped by recombination. During a sequence alignment scan, phylogenetic inhomogeneity is quantified using a quartet mapping approach. Taxa or groups of taxa are subsequently scored using the average inhomogeneity of all the quartets they belong to. To assess whether significantly more inhomogeneity is observed than expected under a recombination-free scenario, we tested MC-simulation, permutation, and a redistribution approach.

Simulation analyses suggested that identifying recombinant sequences without prior specifications is a challenging problem. Only relatively recent recombinants were readily identified in symmetric trees in which recombination was rare. In asymmetric trees, correct recombination identification proved to be more difficult. Exploratory analyses using RDP3 indicated that this was also the case for the triplet-based methods (data not shown). However, the recent recombination event in the asymmetric trees occurred between somewhat more closely related parental strains, with a different mosaic structure, and involved two descendants of the same recombinant (Figure 3). Hence, symmetric and asymmetric tree simulations cannot be easily compared. Asymmetric topologies are generally more problematic in phylogenetic inference; more sequence data is required to recover such topologies and they are much more susceptible to substitution saturation than symmetrical ones [53]. The latter may provide an explanation as to why increased sequence divergence did not result in increased power for recombination detection, as is the case for symmetric trees.

We found marked performance differences between different measures of quartet variability. The poor performance of the total path distance (*d*_*tot*_) may be explained by the fact that the sliding window procedure follows the path between two regions supporting alternative topologies in the quartet-mapping triangle in a stepwise manner, with each step represented by different alignment windows. Steps covering similar distances could also be taken within a region supporting a particular topology, and thus lead to similar total distances in the absence of real topology changes. No matter how many steps need to be taken to the point most distant from the average position, the *d*_*max *_measure only considers how remote this point is from the average positions. The power of the maximum distance or the average distance from the mean position will depend on the mosaic pattern of the recombinant. *d*_*max *_may be more sensitive to a small region with a different evolutionary history, while *d*_*av *_may be more sensitive in case of multiple breakpoints.

The simulation setup was necessarily simplistic and we have not explored the variety of recombination scenarios that occur in reality. When recombination is frequent, for example, all the methods would be expected to suffer from an elevated failure rate [35]. Also the sampling rates of recombinants relative to the parental descendent sequences will have a profound impact on the ability to identify mosaic sequences without prior reference specifications. When few representatives of the recombinant lineage are sampled and relatively closely related descendents of both parents are present, as is the case in our phylogenetic simulations, than the quartet variability should be attributed relatively easily to the recombinant sequences by averaging the *d*_*max *_measure over the quartets that contain these sequences. When, however, many sequences are included that share the same recombination history, more quartets will be composed of multiple recombinant sequences of the same type. These quartets may not generate strong phylogenetic inhomogeneity, and in the case of all four sequences representing the same recombinant, there should be no recombination signal detectable. Although sampling rate inequalities for pre-defined lineages may be controlled to some extent by the grouping constraint we proposed, it will no doubt adversely affect taxa ranking. This may also at least partly explain the poor performance for the asymmetric tree simulations. Here, the more recent recombinant event had two descendents, and frequently, a parental descendent sequence (sequence 3, 4 or 5 in Figure 3) was top ranked instead of one of the recombinants. This again highlights that the current simulations only offer a starting point for comparison of different methods and their relative ability to identify recombinant sequences, and more complexity needs to be explored in the future.

For the quartet scanning method, generating null distributions using MC-simulation was the most powerful procedure for identifying evidence of recombination, but it also suffered from a higher false positive rate in the presence of rate variation among genome regions. The simulated patterns of rate variation are within the range of rate variation that can be expected in HIV genomes. In this case, permutation still achieved good detection rates with far fewer false positives. This difference illustrates that the MC-simulation technique performs very well when the simulated evolution meets the assumptions in the parametric bootstrapping procedure. Under model misspecification, however, it may still be powerful but could also be particularly misleading. Although rate variation can be accommodated in the simulation procedure using a discrete gamma distribution, it assumes that rate variation is independent and identically distributed along the genome. This assumption will rarely be met for real data situations and it can have an important impact on comparative analyses of data partitions across the genome. The redistribution approach, which was designed to homogenize phylogenetic incongruence, but still mimic the pattern of rate variation present in the real data sets, was the least sensitive. Even when rate variation does not correlate with breakpoints, this approach does not easily lead to rejection of clonality. This indicates that permuting sites only with the same entropy score might be too stringent to efficiently homogenize recombination signal. In this respect, it is important to realize that a sequence alignment can include sites with many different entropy values, and the number of sites per entropy value will naturally show a large variation. For a typical simulated data set comprising 16 taxa (symmetric, tree depth = 0.1 substitutions per site, 4000 nucleotides), site patterns can break down in about 46 different site entropy values with, for example, a few patterns having unique entropy values and about 13% of patterns being single nucleotide polymorphisms that are represented by a single entropy value. Therefore, more efficient shuffling under more relaxed rate variation constraints may be achieved by binning site patterns based on their entropy value.

Taxon ranking based on quartet scanning is geared towards the detection of recombinant sequences. As a test for recombination signal, more powerful methods have been devised (see [7] for a comprehensive comparison and, for example, [54]), and we do not provide a formal test for breakpoint locations. Also for the latter, we refer to other developments (e.g. [15] and [39]). However, automated detection of recombinant lineages on its own is already a difficult problem to tackle. The quartet scanning approach performed well relative to various triplet-based methods in identifying recombinants simulated using a phylogenetic approach. However, more comprehensive simulations are needed for a better assessment of the powers and pitfalls in different situations. The essential difference between the quartet scanning approach and the various RDP3 methods does not lie in the use of quartets versus triplets, but how their information is further employed to identify the recombinants. While the quartet approach deduces a ranking for taxa or groups of taxa from the phylogenetic variability in all these quartets, additional tests are used by RDP3 to distinguish recombinants from parental strains in triplets that resulted in significant evidence for recombination. It is interesting to note that the weighting scheme used by RDP3 to combine the results of these tests into a single consensus score is currently based on how accurately the individual tests identified HIV-1M inter-subtype recombinants [35]. Because of the CRF02_AG-subtype G classification artefact, a new weighting optimization might be required. Moreover, quartet scanning might make a useful addition to the tests employed by RDP3.

The quartet approach presented here has particular practical limitations. The number of quartets increases as a fourth order polynomial in the number of taxa, and although the group constraint can alleviate some of the computational burden, both memory requirements and speed of computation will become impractical for very large data sets. This will be a particular burden when many sequential deletions of recombinants are required and significance needs to be assessed each time using replicate data sets. Although these procedures can be automated and quartet information for a permutation alignment can be stored and reused, it would be very useful to be able to test every taxon or taxon group without having to rescan pruned data sets. Our experience with the current implementation leads us to conclude that 50 taxa is roughly the upper limit for this method in practice. There are obviously less triplets than quartets for any data set, and RDP3 does not evaluate evidence for recombination in triplets by analyzing replicate data sets, which makes them faster in computational practice.

In sliding window analyses, it is important to determine how much phylogenetic discordance can be expected from the stochastic nature of the substitution process in the absence of recombination. In BOOTSCAN analysis [10], a query sequence is generally inferred as a recombinant when a high bootstrap support is observed for clustering with different reference sequences in different gene regions. However, this criterion will not be adequate to assess variable clustering deeper in the phylogenetic trees. This is illustrated by the highly divergent HIV-1 variant from the DRC. Although phylogenetic inhomogeneity was originally noted for this strain, the absence of strong bootstrap support for the different clustering deeper in the tree led to the suggestion that it could be a new subtype [50]. However, our test indicates that there is more phylogenetic inhomogeneity than expected by chance and thus classifies the DRC strain as a probable recombinant lineage. Significant phylogenetic incongruence between major genes was also confirmed by various tree topology tests.

Both subtype G in HIV-1 group M and SIVcpz in PIV are confirmatory examples, but the application of quartet scanning demonstrates how recombinants can be easily identified for cases that required in depth analysis to distinguish parental from recombinant lineages [11,52]. The pervasive recombination in PIV lineages has also been suggested by exploratory bootscanning and network analyses [55]. In addition to PIV recombination and HIV-1 intersubtype recombination, our test was also sensitive to intrasubtype recombination. Both in subtype A and D, we identified recombination signals that were supported by network and GARD analyses, and in both cases, the recombination signal did not stem from a single mosaic sequence but from a recombination event that was shared among several sequences.

Although we have identified recombinant genomes at different evolutionary scales in immunodeficiency virus evolution, it may prove a challenging endeavor to clearly delineate breakpoints for many of these recombinants. Similar to other sliding window analyses, breakpoints will be readily identified if viruses from two well-supported phylogenetic clusters recombined relatively recently. However, our method may also pick up recombinants in trees without well-supported groups (e.g. intrasubtype recombinants), ancestral recombinants that were generated before well-supported clades were established (which could be the case for the divergent DRC strain), or ancestral recombinants of lineages that have now diverged considerably (which is probably the case for several PIV lineages).

## Conclusion

In conclusion, we have developed a recombination detection method that does not require *a priori *assignment of reference and query sequences, statistically evaluates the recombination signal in nucleotide and amino acid alignments, and identifies the most plausible recombinants. Taxon ranking and the associated statistical test complement the visual recombination detection method. Collectively, these developments comprise a powerful and versatile methodology that will make a useful addition to the array of recombination detection methods.

## Abbreviations

CRF: circulating recombinant form; PIV: primate immunodeficiency virus; SIV: simian immunodeficiency virus; VisRD: visual recombination detectionl; RDP: Recombination detection program; ME: minimum evolution; F84: Felsenstein '84 model of evolution; GTR: general time-reversible substitution model

## Competing interests

The authors declare that they have no competing interests.

## Authors' contributions

PL conceived of the study, designed the analysis strategy, analyzed the data and drafted the paper. ML carried out the VisRD software implementation and supported data analyses. DM wrote a modified RDP3 version to analyze simulated data and supported data analysis using triplet-based methods and its interpretation. VM conceived of the study, participated in its design and coordination, and helped to draft the manuscript. All authors read and approved the manuscript.

## Supplementary Material

Additional file 1 **Table A1: HIV and SIV Sequence data**. A table listing the accession numbers and subtype/CRF assignment or PIV lineage for the sequences analyzed in this study. Click here for file

Additional file 2 **Coalescent simulations**. This file describes the coalescent simulations and presents the results for different VisRD settings and for a comparison of VisRD to other approaches. Click here for file

Additional file 3 **Additional Table A3**. This table lists the group ranking for quartet scanning of the CRF02_AG data set using *d*_*max*_. Click here for file

Additional file 4 **Additional Table A4**. This table lists the group ranking for quartet scanning of the CRF02_AG data set using *d*_*av*_. Click here for file

Additional file 5 **Additional Table A5**. This table lists the group ranking based on quartet scanning of HIV-1 group M including a divergent DRC lineage. Click here for file

Additional file 6 **Tree reconstructions and topology tests for *gag*, *pol *and *env *data sets including the DRC lineage**. This file includes a figure presenting the results of a phylogenetic analysis for *gag*, *pol *and *env *data sets including the DRC lineage and a table listing tree topology test results for the same data. Click here for file

Additional file 7 **Additional Table A7**. This table lists the group ranking based on quartet scanning of HIV-1 group M including CRF01_AE. Click here for file

Additional file 8 **Additional Table A8**. This table lists the taxon ranking for quartet scanning of the primate immunodeficiency viruses using *d*_*max*_. Click here for file

Additional file 9 **Additional Table A9**. This table lists the taxon ranking for quartet scanning of the primate immunodeficiency viruses using *d*_*av*_. Click here for file
