# Supplementary material for: Identifying recombinants in human and primate immunodeficiency virus sequence alignments using quartet scanning
Source: BMC Bioinformatics. 2009 Apr 27;10:126. doi: 10.1186/1471-2105-10-126 (PMC2684544; doi:10.1186/1471-2105-10-126)
Supplement: Additional file 1 — Table A1: HIV and SIV Sequence data. A table listing the accession numbers and subtype/CRF assignment or PIV lineage for the sequences analyzed in this study. [file 1471-2105-10-126-S1.pdf]

Additional Table A1. HIV and SIV Sequence data.

Database: Accession number	HIV-1 subtype or CRF
GenBank:M62320.1	A1
Genbank:AF286238.1	A2
Genbank:AY521629.1	A3
EMBL:AM000054.1	A4
EMBL:AJ006287.1	B
Genbank:M17449.1	B
Genbank:M17451.1	B
Genbank:U26546.1	B
Genbank:AF286228.1	C
Genbank:DQ396370.1	C
Genbank:AF286234.1	C
Genbank:AF286227.1	C
Genbank:U88824.1	D
Genbank:M27323.1	D
Genbank:K03454.1	D
Genbank:U88822.1	D
Genbank:AF005494.1	F1
EMBL:AJ249238.1	F1
EMBL:AJ249236.1	F2
EMBL:AJ249237.1	F2
Genbank:AF084936.1	G
Genbank:U88826.1	G
Genbank:AF061642.1	G
Genbank:AF061641.1	G
Genbank:AF190127.1	H
Genbank:AF190128.1	H
Genbank:AF005496.1	H
Genbank:AF082395.1	J
Genbank:AF082394.1	J
EMBL:AJ249239.1	K

EMBL:AJ249235.1	K
Genbank:AY125894	CRF01_AE
Genbank:AF197340	CRF01_AE
DDBJ:AB052995	CRF01_AE
Genbank:AY008714	CRF01_AE
Genbank:L39106	CRF02_AG
EMBL:AJ251056	CRF02_AG
Genbank:AY271690	CRF02_AG
Genbank:AF377955	CRF02_AG
Genbank:AF414006	CRF03_AB
Genbank:AF193276	CRF03_AB
Genbank:AF286236	U
Genbank:AF457101	U

Database: Accession number	PIV strain
Genbank: AF103818	SIVcpzUS
Genbank:AF447763	SIVcpzTAN1
Genbank:AF468658	SIVgsn71
Genbank:AF468659	SIVgsn166
Genbank:U72748	SIVsm543
EMBL:X61240	HIV2D205
Genbank:M29975	SIVagm155
Genbank:U58991	SIVagmTAN1
Genbank:AF349680	SIVrcmNigM
Genbank: AF382829	SIVrcmGB1
Genbank:M27470	SIVmndGB1
Genbank:AF301156	SIVcolCGU
Genbank:AF075269	SIVlhoest
Genbank:L06042	SIVsyk
