# Supplementary material for: Identifying recombinants in human and primate immunodeficiency virus sequence alignments using quartet scanning
Source: BMC Bioinformatics. 2009 Apr 27;10:126. doi: 10.1186/1471-2105-10-126 (PMC2684544; doi:10.1186/1471-2105-10-126)
Supplement: Additional file 2 — Coalescent simulations. This file describes the coalescent simulations and presents the results for different VisRD settings and for a comparison of VisRD to other approaches. [file 1471-2105-10-126-S2.pdf]

Coalescent simulations

Simulated data

To evaluate power and false positive rates, we examined simulated data sets previously generated for comparing the performance of 14 recombination detection methods (Posada and Crandall, 2001a). These data sets are now often used to evaluate recombination detection power and false positives of new recombination detection programs (Kosakovsky Pond et al., 2006; Martin et al., 2005). Briefly, 20 groups of 100 10-sequence genealogies were previously generated using a coalescent-based simulation with different recombination rates (recombination parameter ρ = 0, 1, 4, 16, or 64 recombination events in the whole population from which the sample comes from, per site per generation) and different degrees of genetic diversity (θ = 10, 50, 100, or 200 substitutions in the population per site per generation). Ten sequences encompassing 1000 nucleotides were then evolved on the simulated genealogies using the Hasegawa–Kishino–Yano nucleotide substitution model. To assess false positive rates, 16 recombination-free data sets (ρ = 0) have also been simulated previously using the same diversity range but incorporating different degrees of rate heterogeneity among sites (gamma distribution shape parameter $\alpha = \infty$, 2, 0.5, or 0.05). It has been noted that the parameters used for simulation span the range of recombination rates, genetic diversity, and rate heterogeneity typically observed in HIV sequence data from single individuals (Martin et al., 2005; Posada and Crandall, 2001a; Posada and Crandall, 2001b).

Because computation of Monte Carlo *p*-values for all simulated data sets is time consuming, we generate a null distribution using only 20 replicate data sets (instead of 100 in the analysis of real data). Significant recombination signal is inferred when

the mean taxon-ranking values for all the replicate data sets are smaller then the $\bar{r}_t$ for the simulated data set. RECSCAN analysis were performed using RDP3; Simplot and Maximum Chi^2 results were obtained from previously published analyses.

Results

The results are summarized in Fig. A2; each data point in the plots represents the analysis of 100 data sets. For low divergences, there is some difference in recombination detection power between the different approaches to assess significance (Monte Carlo (MC) simulation, permutation and redistribution, see Methods) (Fig. A2a). These differences are less noticeable at higher levels of divergence (e.g., θ = 100, 200). Interestingly, the taxon-ranking test based on any of the null distributions outperforms the widely used SIMPLOT procedure (only taxon ranking based on redistributions is used for comparison in Fig. A2b) (Lole et al., 1999). The performance of our test is also comparable to RECSCAN, although they are both less powerful than the MAXIMUM CHI^2 method (Maynard Smith, 1992), which has been reported as one of the most powerful nonparametric recombination detection methods (Posada and Crandall, 2001a). The RECSCAN results were very similar for either distances or neighbor-joining trees, and only the latter are shown in Fig. A2b. A comparison of different window sizes for the quartet scanning method (200 bp, 350 bp and 500 bp), revealed only relatively small differences at low diversity.

With respect to false positive rates, the taxon-ranking test is generally in the same low range of other methods, like MAXIMUM CHI^2 and SIMPLOT, for different degrees of rate heterogeneity among sites (Fig. A2c). High false positive rates were observed

for taxon-ranking based on MC-simulations applied to data with strong rate heterogeneity (α = 0.05), which is probably due to underestimation of strong rate heterogeneity in the phylogenetic MC-simulation procedure for small-size data sets.

Figure A2. Recombination detection power and false positive rates. (a, b and c) Recombination detection power determined using coalescent-based simulations. Each data point represents the analysis of 100 simulated alignments, 1000 nucleotides in length, evolved under the Hasegawa–Kishino–Yano model of evolution with one of four different degrees divergence (θ = 10, 50, 100, or 200 substitutions in the population per site per generation, indicated using different symbols) and one of five different degrees recombination (ρ = 0, 1, 4, 16, or 64 recombination events in the population per site per generation). A recombination rate of ρ = 0, 1, 4, 16, and 64, respectively, indicates an average of 0, 3, 12, 48, and 192 recombination events in the evolutionary history of each of the alignments examined; two sequences chosen at random from alignments with θ = 10, 50, 100, and 200 are expected to differ at an average of approximately 1%, 5%, 9%, and 17% of their sites, respectively. (a) Comparison of the three different ways to assess significance (MC simulation, permutation and redistribution) for a quartet scanning using a window size of 500 bp. (b) Comparison of quartet scanning (window size of 500 bp, redistributions) with Simplot and MAXIMUM CHI2. (c) Comparison of different window sizes (200 bp, 350 bp, and 500 bp) for the quartet-scanning test based on redistributions. (d) False positive rates determined using datasets simulated without recombination (ρ = 0), but with increasing rate heterogeneity among sites (α = ∞, 2, 0.5 and 0.05). The RECSCAN results are not shown because the false discovery rate was almost consistently 0.

References

Kosakovsky Pond, S. L., Posada, D., Gravenor, M. B., Woelk, C. H., Frost, S. D., 2006. GARD: a genetic algorithm for recombination detection. Bioinformatics 22, 3096-8.

Lole, K. S., Bollinger, R. C., Paranjape, R. S., Gadkari, D., Kulkarni, S. S., Novak, N. G., Ingersoll, R., Sheppard, H. W., Ray, S. C., 1999. Full-length human immunodeficiency virus type 1 genomes from subtype C-infected seroconverters in India, with evidence of intersubtype recombination. J Virol 73, 152-60.

Martin, D. P., Posada, D., Crandall, K. A., Williamson, C., 2005. A modified bootscan algorithm for automated identification of recombinant sequences and recombination breakpoints. AIDS Res Hum Retroviruses 21, 98-102.

Maynard Smith, J., 1992. Analyzing the mosaic structure of genes. J Mol Evol 34, 1369-1390.

Posada, D., Crandall, K. A., 2001a. Evaluation of methods for detecting recombination from DNA sequences: computer simulations. Proc Natl Acad Sci U S A 98, 13757-62.

Posada, D., Crandall, K. A., 2001b. Selecting models of nucleotide substitution: an application to human immunodeficiency virus 1 (HIV-1). Mol Biol Evol 18, 897-906.

Figure A2

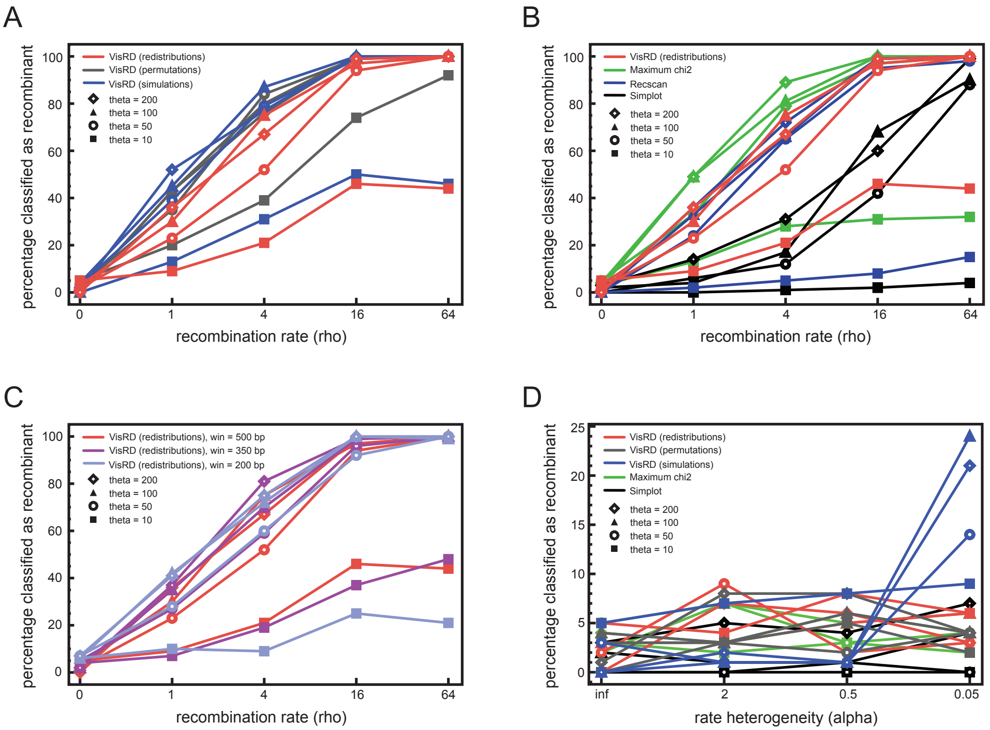
A
VisRD (redistributions)
VisRD (permutations)
VisRD (simulations)
theta = 200
theta = 100
theta = 50
theta = 10
percentage classified as recombinant
recombination rate (rho)
B
VisRD (redistributions)
Maximum chi2
Recscan
Simplot
percentage classified as recombinant
recombination rate (rho)
C
VisRD (redistributions), win = 500 bp
VisRD (redistributions), win = 350 bp
VisRD (redistributions), win = 200 bp
percentage classified as recombinant
recombination rate (rho)
D
VisRD (redistributions)
VisRD (permutations)
VisRD (simulations)
Maximum chi2
Simplot
percentage classified as recombinant
rate heterogeneity (alpha)
