# Supplementary material for: Identifying recombinants in human and primate immunodeficiency virus sequence alignments using quartet scanning
Source: BMC Bioinformatics. 2009 Apr 27;10:126. doi: 10.1186/1471-2105-10-126 (PMC2684544; doi:10.1186/1471-2105-10-126)
Supplement: Additional file 3 — Additional Table A3. This table lists the group ranking for quartet scanning of the CRF02_AG data set using dmax. [file 1471-2105-10-126-S3.pdf]

Additional Table 3. Group ranking for quartet scanning of the CRF02_AG data set using d_{max}.

Group	r_g (%)	Group[1]	r_g (%)	Group[2]	r_g (%)	Group[*]	r_g (%)	Group[3]	r_g (%)	Group[4]	r_g (%)	Group[**]	r_g (%)	Group[5]	r_g (%)	Group[***]	r_g (%)
G (4)	100.0	K (2)	100.0	A (4)	100.0	A1 (1)	100.0	J (2)	100.0	D (4)	100.0	DNDK (1)	100.0	F (4)	100.00	F1BR020 (1)	100.00
CRF02_AG (4)	97.6	A (4)	98.9	D (4)	98.9	A4 (1)	97.6	F (4)	99.6	F (4)	99.8	DELI (1)	99.8	C (4)	99.73	F2MP257 (1)	99.75
J (2)	97.4	D (4)	98.2	J (2)	98.7	A3 (1)	95.8	D (4)	99.5	H (3)	99.6	DZR085 (1)	99.8	H (3)	99.69	F2MP255 (1)	99.74
A (4)	96.0	J (2)	98.0	C (4)	98.2	D (4)	95.3	H (3)	98.9	A2 (1)	99.0	F (4)	98.9	CRF02_AG (4)	99.32	F1MP411 (1)	97.09
K (2)	95.3	F (4)	97.7	CRF02_AG (4)	98.2	J (2)	94.7	C (4)	98.7	C (4)	99.0	H (3)	98.4	B (4)	99.25	H (3)	96.66
F (4)	94.8	C (4)	97.3	F (4)	98.0	A2 (1)	94.5	A2 (4)	98.3	CRF02_AG (4)	98.7	A2 (1)	98.0	DUG114 (1)	98.66	C (4)	96.10
D (4)	94.3	CRF02_AG (4)	97.2	B (4)	97.4	C (4)	94.4	B (4)	98.0	B (4)	98.3	C (4)	97.8	A2 (1)	98.44	CRF02_AG (4)	95.66
H (3)	93.0	B (4)	96.6	H (3)	97.2	CRF02_AG (4)	94.4	CRF02_AG (4)	97.8			CRF02_AG (4)	97.4			A2 (1)	95.62
C (4)	92.5	H (3)	96.2			F (4)	94.1					B (4)	97.0			B (4)	95.49
B (4)	92.4					B (4)	93.8					DUG114 (1)	95.5			DUG114 (1)	94.76
						H (3)	93.4										

The number of sequences per group is indicated between brackets. [1-5] The group ranking based on d_{max} was generated after sequential exclusion of the subtype G sequences[1], subtype K sequences[2], subsubtype A1, A3 and A4 sequences[3], subtype J sequences[4] and subtype D sequences[5] (NDK, ELI and ZR085). [*,**,***] Group ranking after removing the grouping constraint for subtype A[*], subtype D[**] And subtype F[***]. All permutation p-values were < 0.01; after excluding the subtype F sequences no significant recombination was detected ($p = 0.19$)
