# Supplementary material for: Identifying recombinants in human and primate immunodeficiency virus sequence alignments using quartet scanning
Source: BMC Bioinformatics. 2009 Apr 27;10:126. doi: 10.1186/1471-2105-10-126 (PMC2684544; doi:10.1186/1471-2105-10-126)
Supplement: Additional file 4 — Additional Table A4. This table lists the group ranking for quartet scanning of the CRF02_AG data set using dav. [file 1471-2105-10-126-S4.pdf]

Additional Table A4. Group ranking for quartet scanning of the CRF02_AG data set using d_{av}.

Group	r_g (%)	Group[1]	r_g (%)	Group[2]	r_g (%)	Group[*]	r_g (%)	Group[3]	r_g (%)	Group[4]	r_g (%)	Group[**]	r_g (%)	Group[5]	r_g (%)	Group[***]	r_g (%)
G (4)	100.0	K (2)	100.0	A (4)	100.0	A1 (1)	100.0	D (4)	100.0	DNDK (1)	100.0	DZR085 (1)	100.0	F (4)	100.0	F2MP257 (1)	100.0
CRF02_AG (4)	98.0	A (4)	99.3	D (4)	98.0	A3 (1)	97.2	F (4)	99.8	DELI (1)	99.4	J (2)	99.4	H (3)	99.6	F1BR (1)	98.4
J (2)	96.0	D (4)	98.0	CRF02_AG (4)	98.0	A4 (1)	96.4	H (3)	99.6	F (4)	99.1	F (4)	99.2	J (2)	98.7	F2MP255 (1)	98.1
A (4)	95.2	CRF02_AG (4)	97.81	F (4)	97.3	D (4)	94.2	B (4)	99.1	DZR085 (1)	98.9	H (3)	98.4	B (4)	98.6	F1MP4 (1)	97.3
K (2)	94.4	F (4)	97.1	C (4)	97.0	CRF02_AG (4)	93.8	J (2)	98.9	H (3)	98.4	C (4)	98.4	DUG114 (1)	98.4	J (2)	96.6
F (4)	93.2	C (4)	96.6	B (4)	96.7	F (4)	93.3	A2 (1)	98.7	A2 (1)	98.0	B (4)	98.1	A2 (1)	98.2	H (3)	96.4
D (4)	93.1	B (4)	96.5	H (3)	96.6	C (4)	93.3	C (4)	98.4	B (4)	97.9	CRF02_AG (4)	97.6	C (4)	98.1	C (4)	95.6
H (3)	92.2	J (2)	96.4	J (2)	96.1	A2 (1)	93.3	CRF02_AG (4)	97.4	J (2)	97.8	A2 (1)	97.5	CRF02_AG (4)	98.0	A2 (1)	95.3
B (4)	91.7	H (3)	95.6			B (4)	93.1			C (40	97.5	DUG114 (1)	95.9			B (4)	95.0
C (4)	91.6					H (3)	92.8			CRF02_AG (4)	96.5					CRF02_AG (4)	94.8
						J (2)	92.4			DUG114 (1)	96.2					DUG114 (1)	93.6

The number of sequences per group is indicated between brackets. [1-5] The group ranking based on d_{av} was generated after sequential exclusion of the subtype G sequences[1], subtype K sequences[2], subsubtype A1, A3 and A4 sequences[3] and subtype D sequences[4,5] (DNDK, DELI and DZR085). [*,**,***] Group ranking after removing the cluster constraint for subtype A[*], subtype D[**] And subtype F[***]. All permutation p-values were < 0.01; after excluding the subtype F sequences no significant recombination was detected ($p = 0.08$).
