# Supplementary material for: Identifying recombinants in human and primate immunodeficiency virus sequence alignments using quartet scanning
Source: BMC Bioinformatics. 2009 Apr 27;10:126. doi: 10.1186/1471-2105-10-126 (PMC2684544; doi:10.1186/1471-2105-10-126)
Supplement: Additional file 5 — Additional Table A5. This table lists the group ranking based on quartet scanning of HIV-1 group M including a divergent DRC lineage. [file 1471-2105-10-126-S5.pdf]

Table A5. Group ranking based on quartet scanning of HIV-1 group M including a divergent DRC lineage.

Groups[4]	r_g (%)
DRC_U (2)	100.0
A2 (1)	99.7
C (4)	99.5
B (4)	99.2
H (3)	98.2
CRF02_AG (4)	97.9
DUG114 (1)	97.4

The number of sequences per group is indicated between brackets. The permutation *p*-value < 0.01 was based on d_{max}.
