# Supplementary material for: Identifying recombinants in human and primate immunodeficiency virus sequence alignments using quartet scanning
Source: BMC Bioinformatics. 2009 Apr 27;10:126. doi: 10.1186/1471-2105-10-126 (PMC2684544; doi:10.1186/1471-2105-10-126)
Supplement: Additional file 6 — Tree reconstructions and topology tests for gag, pol and env data sets including the DRC lineage. This file includes a figure presenting the results of a phylogenetic analysis for gag, pol and env data sets including the DRC lineage and a table listing tree topology test results for the same data. [file 1471-2105-10-126-S6.pdf]

**File 6. Tree reconstructions and topology tests for *gag*, *pol* and *env* data sets including the DRC lineage.**

Fig. A6. HIV-1 Maximum likelihood trees for the structural genes *gag*, *pol* and *env*. The numbers at the nodes represent the bootstrap support percentages based on 100 replicates. The DRC sequences are indicated with a rectangle.

Table A6. *p*-values for one-sided Kishino-Hasegawa test, the Shimodaira-Hasegawa test and the approximately unbiased test.

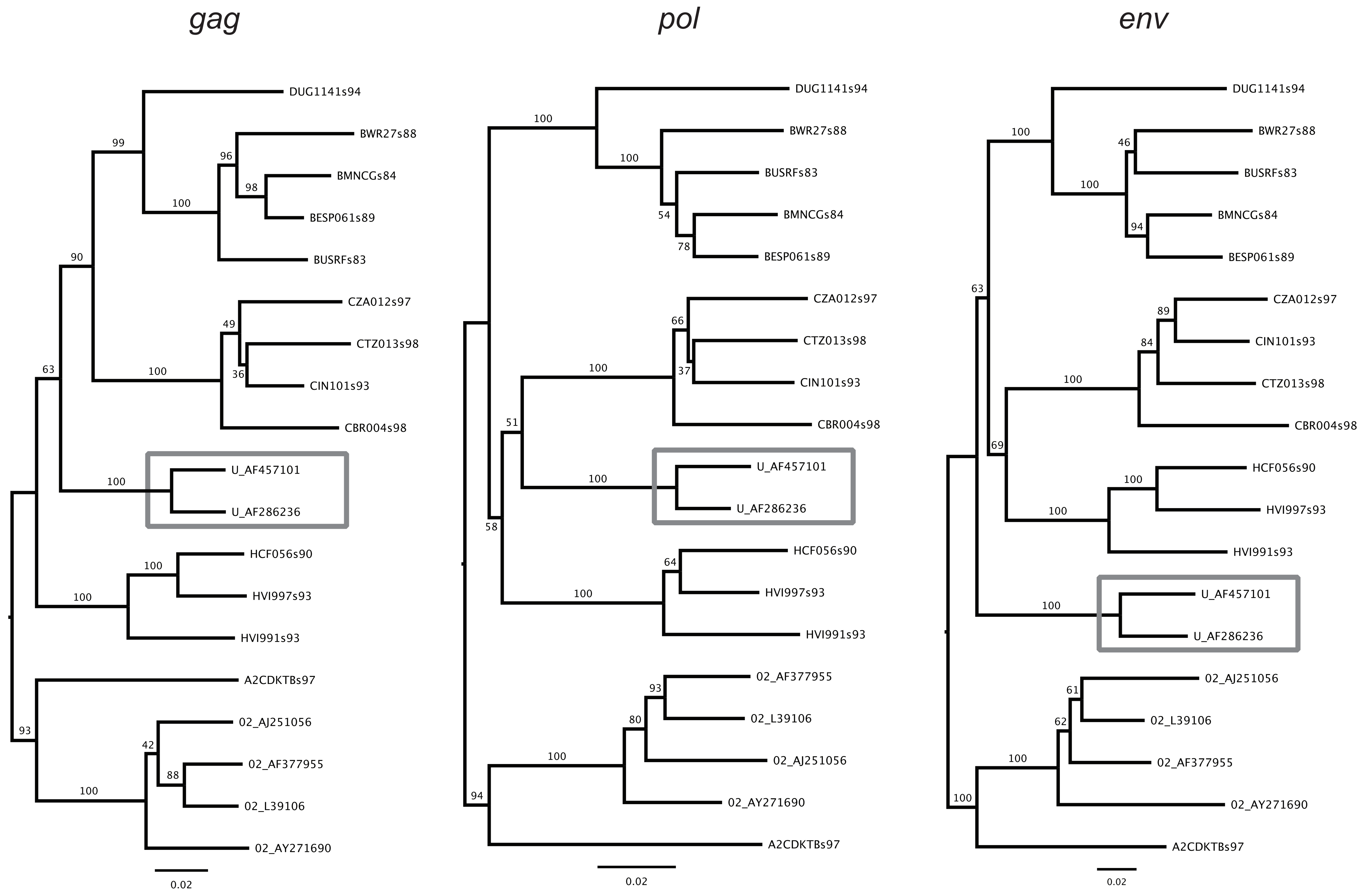
gag
pol
env
DUG1141s94
BWR27s88
BMNCGs84
BESP061s89
BUSRFs83
CZA012s97
CTZ013s98
CIN101s93
CBR004s98
U_AF457101
U_AF286236
HCF056s90
HVI997s93
HVI991s93
A2CDKTBs97
02_AJ251056
02_AF377955
02_L39106
02_AY271690
0.02

Table A6. *p*-values for tree incongruence tests

| | Data | | | | | | | | |
|---|---|---|---|---|---|---|---|---|---|
| | gag | | | pol | | | env | | |
| Tree | KH | SH | AU | KH | SH | AU | KH | SH | AU |
| gag | (Best ML tree) | | | 0.208 | 0.329 | 0.228 | 0.025* | 0.026* | 0.035* |
| pol | 0.006** | 0.008** | 0.002** | (Best ML tree) | | | 0.032* | 0.021* | 0.027* |
| env | 0.035* | 0.039* | 0.048* | 0.026* | 0.030* | 0.017* | (Best ML tree) | | |

KH, one-sided Kishino-Hasegawa test; SH, Shimodaira-Hasegawa test; AU, approximately unbiased test. * significant at the 0.05 level; ** significant at the 0.01 level
