# Supplementary material for: Identifying recombinants in human and primate immunodeficiency virus sequence alignments using quartet scanning
Source: BMC Bioinformatics. 2009 Apr 27;10:126. doi: 10.1186/1471-2105-10-126 (PMC2684544; doi:10.1186/1471-2105-10-126)
Supplement: Additional file 7 — Additional Table A7. This table lists the group ranking based on quartet scanning of HIV-1 group M including CRF01_AE. [file 1471-2105-10-126-S7.pdf]

Table A7. Group ranking based on quartet scanning of HIV-1 group M including a CRF02_AE.

Groups[4]	r_g (%)
CRF02_AE (4)	100.0
CRF02_AG (4)	99.4
B (4)	94.9
C (4)	94.8
A2 (1)	92.0
DUG114 (1)	92.0
H (3)	89.9

The number of sequences per group is indicated between brackets. The permutation p-value < 0.01 was based on d_{max}.
