# Supplementary material for: Identifying recombinants in human and primate immunodeficiency virus sequence alignments using quartet scanning
Source: BMC Bioinformatics. 2009 Apr 27;10:126. doi: 10.1186/1471-2105-10-126 (PMC2684544; doi:10.1186/1471-2105-10-126)
Supplement: Additional file 8 — Additional Table A8. This table lists the taxon ranking for quartet scanning of the primate immunodeficiency viruses using dmax. [file 1471-2105-10-126-S8.pdf]

Additional Table A8. Taxon ranking for quartet scanning of primate immunodeficiency viruses using d_{max}.

Taxa	r_t (%)	Taxa[1]	r_t (%)	Taxa[2]	r_t (%)	Taxa[3]	r_t (%)	Taxa[4]	r_t (%)	Taxa[5]	r_t (%)
SIVcpzUS	100.0	SIVagm155	100.0	SIVcolCGU	100.0	SIVmndGB1	100.0	SIVsyk173	100.0	SIVsm543	100.0
SIVcpzTan1	99.2	SIVagmTAN1	96.5	SIVmndGB1	98.3	SIVsyk173	99.3	SIVsm543	94.9	HIV2D205	97.7
SIVagm155	96.3	SIVmndGB1	95.0	SIVsm543	96.2	SIVsm543	99.1	HIV2D205	94.6	SIVrcmNigM	94.0
SIVagmTAN1	95.5	HIV2D205	94.8	SIVlhoest7G	96.2	SIVlhoest7G	98.4	SIVrcmGB1	90.0	SIVrcmGB1	90.6
SIVsm543	93.9	SIVsm543	94.3	SIVsyk173	96.0	SIVrcmNigM	98.3	SIVrcmNigM	88.9	SIVgsn71	85.5
SIVmndGB1	93.8	SIVcolCGU	94.2	HIV2D205	95.0	HIV2D205	97.7	SIVgsn71	86.0	SIVgsn166	84.1
SIVrcmNigM	93.8	SIVsyk173	94.1	SIVrcmNigM	91.6	SIVrcmGB1	93.3	SIVgsn166	82.2		
HIV2D205	93.5	SIVlhoest7G	92.1	SIVrcmGB1	90.0	SIVgsn71	88.5				
SIVsyk173	92.9	SIVrcmNigM	88.5	SIVgsn71	87.3	SIVgsn166	85.5				
SIVrcmGB1	92.7	SIVrcmGB1	87.3	SIVgsn166	85.2						
SIVlhoest7G	91.6	SIVgsn71	85.6								
SIVcolCGU	89.9	SIVgsn166	85.1								
SIVgsn71	88.9										
SIVgsn166	88.4										

[1-5] The taxon ranking based on d_{max} was generated after sequential exclusion of SIVcpzUS and SIVcpzTan1[1], SIVagm155 and SIVagmTAN1[2], SIVcolCGU[3], SIVmndGB14 and SIVlhoest7G[4] (although SIVlhoest7G was not immediately ranked after SIVmndGB14, it was the top ranked taxon after deletion of SIVmndGB14), SIVsyk173[5]. All permutation *p*-values were < 0.01.
