# Supplementary material for: Identifying recombinants in human and primate immunodeficiency virus sequence alignments using quartet scanning
Source: BMC Bioinformatics. 2009 Apr 27;10:126. doi: 10.1186/1471-2105-10-126 (PMC2684544; doi:10.1186/1471-2105-10-126)
Supplement: Additional file 9 — Additional Table A9. This table lists the taxon ranking for quartet scanning of the primate immunodeficiency viruses using dav. [file 1471-2105-10-126-S9.pdf]

Additional Table A9. Taxon ranking for quartet scanning of primate immunodeficiency viruses using d_{av}.

Taxa	r_t (%)	Taxa[1]	r_t (%)	Taxa[2]	r_t (%)	Taxa[3]	r_t (%)	Taxa[4]	r_t (%)	Taxa[5]	r_t (%)
SIVcpzTan1	100.00	SIVagmTAN1	100.00	SIVmndGB1	100.00	SIVsyk173	100.00	SIVcolCGU	100.00	HIV2D205	100.00
SIVcpzUS	98.17	SIVagm155	99.40	SIVlhst7G	99.04	SIVcolCGU	99.73	HIV2D205	94.00	SIVsm543	99.77
SIVagmTAN1	92.48	SIVmndGB1	98.58	SIVcolCGU	97.86	SIVsm543	93.99	SIVsm543	93.39	SIVrcmNigM	96.90
SIVagm155	91.84	SIVcolCGU	95.70	SIVsyk173	96.86	HIV2D205	92.27	SIVrcmGB1	92.32	SIVrcmGB1	95.69
SIVrcmNigM	90.66	SIVlhoest7G	95.41	SIVsm543	94.35	SIVrcmGB1	89.87	SIVrcmNigM	91.73	SIVgsn166	85.40
SIVmndGB1	90.55	SIVsyk173	95.27	SIVrcmNigM	92.62	SIVrcmNigM	89.52	SIVgsn166	81.75	SIVgsn71	83.86
SIVsyk173	89.93	SIVsm543	94.39	HIV2D205	91.82	SIVgsn71	84.15	SIVgsn71	79.57		
SIVsm543	88.77	HIV2D205	92.04	SIVrcmGB1	88.57	SIVgsn166	82.97				
SIVlhoest7G	88.37	SIVrcmNigM	90.06	SIVgsn71	84.91						
SIVrcmGB1	88.04	SIVrcmGB1	86.33	SIVgsn166	84.04						
HIV2D205	87.51	SIVgsn71	84.03								
SIVcolCGU	86.78	SIVgsn166	83.50								
SIVgsn71	86.43										
SIVgsn166	86.34										

[1-5] The taxon ranking was generated after sequential exclusion of SIVcpzUS and SIVcpzTan1[1], SIVagmTAN1 and SIVagm155[2], SIVmndGB14 and SIVlhoest7G[3], SIVsyk173[4] and SIVcolCGU[5]. All p-values were < 0.01; after excluding the subtype SIVsm/HIV-2(D205) sequences, no significant recombination was detected ($p = 0.16$).
